# Creating more and more new institutions may not make the world safer from pandemics

**DOI:** 10.1371/journal.pgph.0001921

**Published:** 2023-05-17

**Authors:** Clare Wenham

**Affiliations:** Department of Health Policy, London School of Economics (LSE), London, United Kingdom; PLOS: Public Library of Science, UNITED STATES

In the three years since the emergence of COVID-19, we have witnessed the explosion of reviews to assess what went wrong with the response, and mechanisms to readdress such failures have been established. At the level of global governance, all the usual actors are involved: The World Health Organization has begun the IHR Amendment Process [1] and the development of a Pandemic Accord (CA+) [2]. The World Bank created a Pandemic Fund [3], and the United Nations General Assembly is hosting a high-level meeting in September 2023 to consider pandemic preparedness and response [4]. More recently, a multi-stakeholder platform for equitable access to medical countermeasures has been announced [5]. Some of these institutions and programmes have developed from contemporary working efforts, such as those of COVID-19 related working groups amid the G20, G8, and the Independent Panel for Pandemic Preparedness and Response [6]. Moreover, COVID-19 has led to new actors getting involved in global health security debates that previously were not, such as the IMF. So where does this leave pandemic preparedness and response, and global health, as a whole? Quite simply: in a mess amid a chaotic landscape of geopolitical chess.

This proliferation of new actors and processes is not new in global health–it mimics the so-called “golden-years” of the early 2000s in global health where we witnessed the creation of multiple new institutions (e.g. BMGF, Global Fund, Gavi, Unitaid) [7]. These were borne from a frustration of antiquated and slow-paced models of governance, or to provide new mechanisms to facilitate the inclusion of a broader array of entities and financing models. This ranged from states, state-related initiatives, private sector, public-private partnerships, philanthropies, NGOs, and the variety of interactions between these [8]. The diversification of the centre of gravity in global health gave power to actors such as the Bill & Melinda Gates Foundation, paving the way for non-state agencies to play a big role in shaping policy.

In many ways, this period of global health created a precedent for a path dependency of new institutions being created in the sector, either as add-ons to existing organizations, as hybrid creatures between institutions, or as new establishments. A path dependency characterised by power struggles amid different states.

The absurdity of many these numerous new institutions for health security is that they are all ostensibly seeking to do the same thing: to make the world more prepared for a future health emergency, and mitigate its effects it if does emerge. Moreover, many of them have the very same membership, whether as a group of individuals or as member states that negotiate the governance, finances, mandate and policies. So why do we see this proliferation of actors if they are the same people, considering the same issues, mostly in the same city? This is not by accident, but reflects the very real political game playing that occurs within multilateralism.

Within international relations such a phenomenon is not new–and can be called many different things depending on one’s preponderance for cynicism: forum shifting [9], forum shopping [10], counter-institutionalization [11], regime complexes, layering or even reflecting coexisting path dependencies [12]. The outcome is the same: rich countries are trying to move the location of policy development and negotiation from one location of governance to another location in the search of more favourable outcomes. This is ostensibly done to ensure greater power in setting the agenda, or deciding on policy in their terms on the basis of governance characteristics of a particular venue, membership, mandate, decision making processes or enforcement options [10]. What’s more, among themselves, high income countries are seeking to play geopolitics within the global multilateral system to get controlling stakes in the process and policies to be shaped for pandemic preparedness and response within their particular world view and to better serve their interests.

This is exactly what we are seeing in contemporary global health security. For example, the US government has pushed governance reform through the International Health Regulations (IHR) amendments [1], a mechanism which has no enforcement optionality to chastise them in future for failure to comply. At the same time, they have facilitated the operationalisation of health security through support of the Pandemic Fund, noting the greater voting rights it has at the World Bank, and therefore the US has disproportionate influence in policy development. In a counter move, the European Union sought to create the Pandemic CA+, a new treaty or accord over which they hoped to lead the conversation in creating the definitive political commitment to future pandemics, and to do so in a new forum away from the failures of the IHR. They hoped this would bring a new holistic approach to pandemic preparedness, including recognition of health systems, One Health and the health workforce [2]. Meanwhile LMICs think both of these measures fail to address the elephant in the room, which is equitable access to vaccines and other medical countermeasures, which, three years into the pandemic, is something they are still fighting for. Given that amid WHO governance, there is one state one vote, there has been a recent move by richer countries to move this question of vaccine equity to a new multilateral platform for equitable access. This has notably established by those very governments that blocked the TRIPS waiver for intellectual property access for COVID-19 vaccines during COVID, and can be seen to be moving the question of equity in terms of access to vaccines away from the auspices of a legally binding accord under which governments could be held to account for their actions.

Meanwhile, amid the global level scrappy fighting of global geopolitics within the health arena, regional actors increasingly are turning to regional solutions, seeing that global multilateral systems failed them during the COVID pandemic and do not seem likely to do much better in the future. This trend is not new within health multilateralism, noting the strong role that WHO regional offices play [12], but such regionalism is now expanding beyond WHO to the rest of global health security. For example, on the African continent, the African Union created the African CDC and a number of regional and sub-regional bodies to provide a series of pandemic preparedness and response functions [13], part of a trend of self-determination and African institutionalism [14]. Similarly, the EU has created HERA, and the institution is increasingly becoming an important actor in global health governance in its own right [15]. These join ASEAN and Mercosur in the global health governance landscape, each offering further vehicles for more favourable negotiations at different levels of governance.

The multiple streams that are occurring seem to use the ubiquitous buzz words of “synergy” and “interconnectedness”, but none have yet to take meaningful steps to achieving this. Within WHO itself, the two processes of the IHR Amendments and Pandemic CA+ are due to be delivered in May 2024, meaning that there is a risk that clauses will be negotiated out of one process and not picked up in the other due to a lack of sequencing oversight. Similarly, the Pandemic Fund has launched a call for proposals, but this is out of sync with WHO processes, and may be affected by any IHR amendments, as it uses an associated proposed results framework. Thus, the irony is that the very thing that all these processes are trying to address is being weakened by the game playing by governments in their negotiation venues and processes. Or, perhaps this is not ironic at all, but is simply global politics. Yet, we must recognise that multiple competing processes will not leave the world safer from the threat of pandemics, but will create a patchwork of semi-aligned institutions and policies which will likely lead to greater fragmentation both within the health sector, and geopolitically. The problem is what to do about it: governments will be governments, and will not simply coordinate and offer equitable solutions because those working in public health think it’s best. We must recognise such political game playing, and develop strategies to work amid these institutional developments, rather than pretend it is not happening.
